# Kisspeptin and Polycystic Ovary Syndrome

**DOI:** 10.3389/fendo.2019.00298

**Published:** 2019-05-10

**Authors:** Rong Tang, Xiaohong Ding, Jianghu Zhu

**Affiliations:** ^1^Department of Pediatrics, The Second Affiliated Hospital of Wenzhou Medical University, Wenzhou, China; ^2^The Second School of Medicine, Wenzhou Medical University, Wenzhou, China; ^3^The First Clinical Medical School, Wenzhou Medical University, Wenzhou, China; ^4^Key Laboratory of Obstetric and Gynecologic and Pediatric Diseases and Birth Defects, Ministry of Education, West China Second University Hospital, Sichuan University, Chengdu, China

**Keywords:** kisspeptin, polycystic ovary syndrome, reproduction, hypothalamus, metabolism

## Abstract

Although the pathogenesis of Polycystic Ovary Syndrome (PCOS) is still unclear, the disturbance of hypothalamic-pituitary-gonadal (HPG) axis is suspected to be the main culprit in the development of PCOS. Kisspeptin, a hypothalamic peptide encoded by the KISS1 gene, is widely reported as a key factor in the regulation of luteinizing hormone (LH)/ follicular-stimulating hormone (FSH) secretion, which may be potentially involved with the development of PCOS.

**Objective:** The objective of this study is to summarize the existing knowledge in the literature in terms of the circulating kisspeptin concentration in PCOS women, kisspeptin and metabolic profiles in PCOS women and kisspeptin expression in PCOS animal models.

**Method:** A systematic literature search was conducted using “Pubmed,” “Embase,” “Web of Science” for all English language articles published up to July 2018 with the terms “PCOS,” “Stein-Leventhal Syndrome,” “Polycystic ovary syndrome,” “metastins” and “kisspeptin”.

**Conclusion:** Overall, kisspeptin levels are higher in the PCOS population, which supports the hypothesis that an over-active KISS1 system leads to enhanced HPG-axis activity, thereby causing irregular menstrual cycles and excessive androgen release in PCOS women.

## Introduction

Polycystic ovary syndrome (PCOS) is a common endocrine and metabolic disorder, which is characterized by chronic anovulation, polycystic ovaries and hyperandrogenism (1), affecting 5–22% of women of reproductive age (2). Although the pathogenesis of PCOS is still unclear, the disturbance of the hypothalamic-pituitary-gonadal (HPG) axis, featuring elevated luteinizing hormone (LH)/ follicular-stimulating hormone (FSH) ratios, is strongly suspected to be associated with the development of PCOS in about 35-90% patients (3–6). However, the mechanism involved with the dysregulated HPG axis in PCOS has not been well-illustrated yet.

Kisspeptin is a hypothalamic peptide encoded by the KISS1 gene, which was first isolated from the human placenta (7). Since it was discovered, substantial studies, based on the level of cell, animal model and even human-beings, reported a key role of kisspeptin in regulation of the HPG axis (8–12). Some studies observed that the administration of kisspeptin could lead to an over 2-fold increase in LH level, accompanied by a small or non-existent elevation of FSH level (13–15). Meanwhile, several researchers noted that kisspeptin exerts a direct effect on the upstream of GnRH neurons in terms of depolarization, higher firing rate and the up-regulated expression of GnRH mRNA, which explains the increased LH/FSH ratio after kisspeptin administration observed in those previous reports (16–19). Although the presence of kisspeptin receptors on pituitary has been reported, these receptors seemed to have little stimulatory effect on gonadotropin release when GnRH antagonist was applied, suggesting that the direct effect of kisspeptin on GnRH neurons is the major pathway (20, 21).

Although the above studies have unveiled the potential correlation between the KISS1 system and the HPG axis, whether the plasma/serum kisspeptin concentration is higher in PCOS women compared with general population remains inconclusive. Some studies observed a higher level of kisspeptin in PCOS women than controls (22, 23), while other studies showed comparable or negatively-correlated results (24, 25). However, according to the existing evidence of kisspeptin in regulating the HPG axis, it is biologically plausible that the number of kisspeptin neurons may be positively correlated with LH level (26). Therefore, plasma/serum kisspeptin levels can also be expected to be positively associated with serum LH level, although whether circulating kisspeptin levels reflect the number of kisspeptin neurons in the hypothalamus is not known.

**Figure 1:**
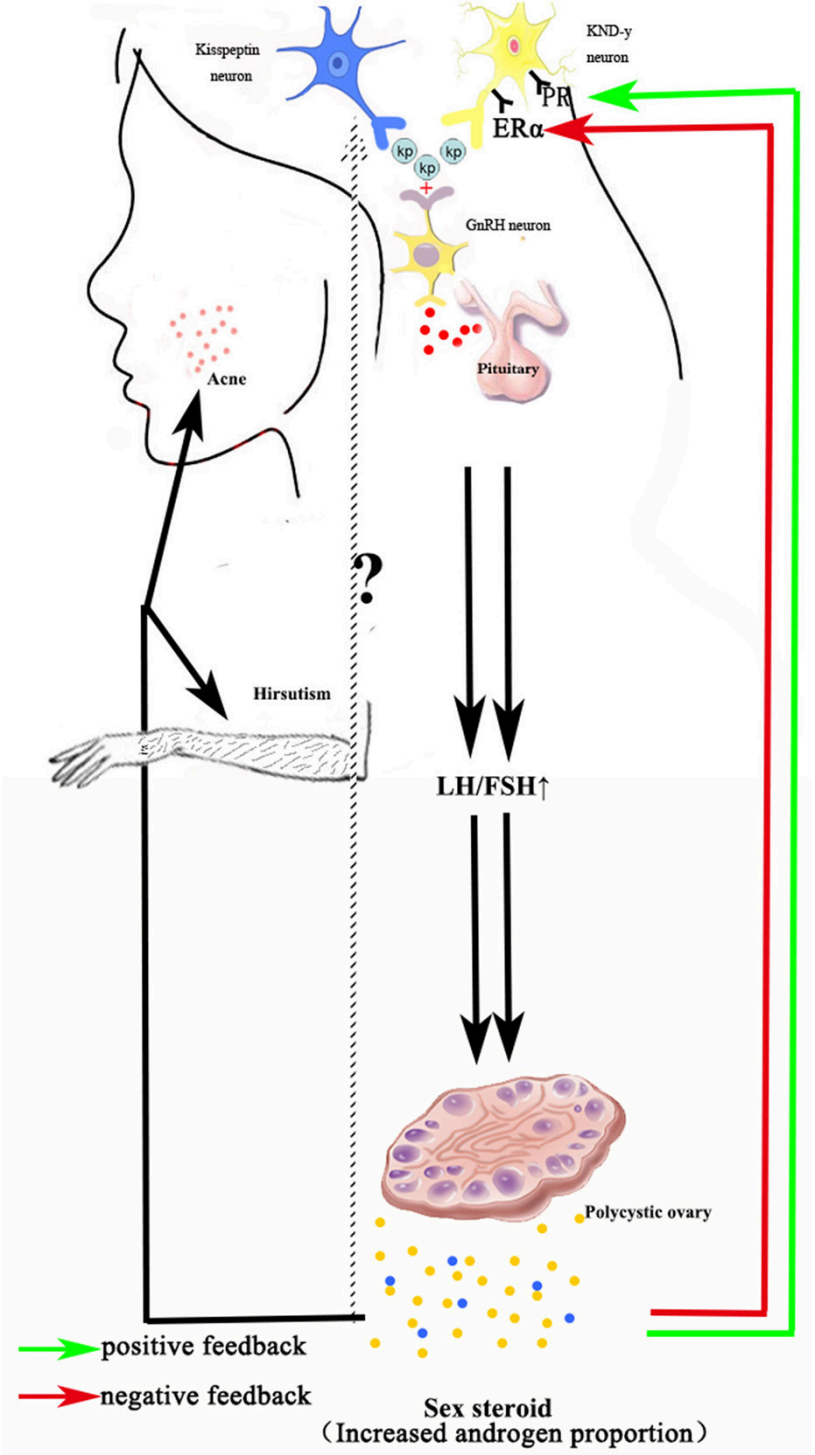
Schematic diagram depicting how kisspeptin involved with PCOS. Both kisspeptin neuron and KND-y neuron can generate and release kisspeptin, which binds with the receptors expressed by GnRH neuron, facilitating the release of GnRH. Therefore, over-expressed kisspeptin is expected to cause an enhanced HPG-axis activity, leading to a higher LH/FSH ratio and excessive androgen secretion, which disturbs the function and morphology of ovary and making acne and hirsutism more susceptible. KND-y neuron, Kisspeptin-Neurokinin B-Dynorphin Neuron; kp, Kisspeptin; GnRH, Gonadotropin-Releasing Hormone; LH, Luteinizing Hormone; FSH, Follicular-stimulating Hormone; PR, Progesterone receptor; ERα, Estrogen receptor-α. 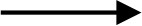
Feedback of sex steroid for KND-y neuron has been confirmed. 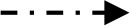
Feedback of steroid for Kisspeptin neuron has not been confirmed yet.

Thus, it is necessary to summarize the existing knowledge with regards to the relationship between PCOS and kisspeptin in the literature and review whether circulating kisspeptin concentrations are increased in women with PCOS. Figure out the underlying role of kisspeptin involved with the pathogenesis of PCOS may provide valuable information and directions for future studies (Figure 1).

## Method

A systematic literature search was conducted using “Pubmed,” “Embase,” “Web of Science” for all English language articles published up to July 2018 with the terms “PCOS,” “Stein-Leventhal Syndrome,” “Polycystic ovary syndrome,” “metastins” and “kisspeptin”. The final item for retrieve is “(((Stein-Leventhal Syndrome) [Title/Abstract] OR (PCOS) [Title/Abstract] OR (Polycystic Ovarian Syndrome) [Title/Abstract])) AND ((Metastins[Title/Abstract] OR kisspeptin)[Title/Abstract])”.

## Kisspeptin Levels in Women With PCOS

Since Panidis et al firstly compared the kisspeptin level between PCOS women and non-PCOS females (24), a total of 12 studies were subsequently conducted during the last decade, contributing to investigate the relationship between plasma/serum kisspeptin level and the presence of PCOS (shown in Table 1). It was biologically plausible that the kisspeptin level should be higher in PCOS women according to previously *in vivo*/*vitro* findings (8–12). As expected, eight out of twelve studies, to the best of our knowledge, have reported a higher kisspeptin level in PCOS women compared with controls. Among the other four studies, Albalawi et al did not keep the comparability of age and BMI between PCOS and non-PCOS group, and they only considered “oligomenorrhoea” and “abnormal ovary” into the diagnosis criteria of PCOS (25). Similarly, Panidis et al only considered “Chronic anovulation” and “hyperandrogenemia” as the diagnostic norm when they enrolled participants in their trials (24). Actually, different PCOS phenotypes are characterized by various metabolic features, raising the possibility that the kisspeptin level may not be increased in all subtypes. A high proportion of one PCOS phenotype with normal kisspeptin levels potentially distorts the measured kisspeptin concentration in the whole sample, which could partially explain the negative findings reported in some studies. However, while some rodents studies provided persuasive evidence, none of the existing studies has exclusively evaluated the kisspeptin level in each subtype of PCOS patients. The other reason to explain the negative finding in some studies may be the small sample size, varying from only 58 to 400 patients, which is susceptible to cause unexpected sampling error. Actually, aside from hypothalamic overactivity, some other factors such as pituitary and ovarian dysfunction may also contribute at least partially to the development of PCOS. For some PCOS patients, hypothalamic overactivity might be less severe than ovarian dysfunction, while the role of kisspeptin in the ovary has rarely been reported previously and is seemingly insignificant, which explains why these patients show PCOS symptoms, but elevated kisspeptin levels are not detected in their blood. In conclusion, while this review stated kisspeptin levels are higher in women with PCOS compared with controls according to qualitative literature-based evidence, the theory that not all PCOS subtypes could show higher kisspeptin levels should be investigated in future studies.

Table 1Comparison of kisspeptin level between PCOS women and control group.

**Table:** 

**Reference**	**PCOS diagnosis criteria**	**Age(PCOS vs. control) (y)**	**BMI(PCOS vs. control) (kg/m^**2**^)**
Emekci Ozay, et al. (26)	Rotterdam	23.99 ± 4.63 vs. 24.43 ± 4.39	24.32 ± 3.40 vs. 23.44 ± 4.08
Albalawi et al. (25)	Oligomenorrhoea and abnormaL ovary	29.4 ± 3.93 vs. 26.7 ± 3.6^*^	46.04 ± 7.3 vs. 36.6 ± 8.17^*^
Chen et al. (22)	Rotterdam	18.6 ± 0.68 vs. 17.89 ± 1.24	20.11 ± 2.31 vs. 21.46 ± 4.60
Daghestani (27)	Rotterdam	24.05 ± 4.41 vs. 25.43 ± 4.80	22.84 ± 1.52 vs. 22.12 ± 1.48
Gorkem et al. (23)	Rotterdam	27.76 ± 4.65 vs. 27.92 ± 4.67	26.34 ± 4.69 vs. 26.06 ± 5.39
Jeon et al. (28)	Rotterdam	23.88 ± 4.86 vs. 24.92 ± 2.94	20.23 ± 2.19 vs. 19.77 ± 1.51^*^
Nyagolova et al. (29)	Rotterdam	24.99 ± 0.49 vs. 26.65 ± 0.71	25.59 ± 0.65 vs. 24.88 ± 0.89
Panidis et al. (24)	Chronic anovulation and hyperandrogenemia	24.30 ± 1.18 vs. 26.85 ± 1.06	33.02 ± 0.56 vs. 32.13 ± 1.85
Yilmaz et al. (30)	Rotterdam	21 (17-25, 25-36) vs. 24.5 (17-25, 25-37)^*^	24.5 (18.1–34.8) vs. 23.1 (18–34.8)
Kaya et al. (31)	Rotterdam	28.75 ± 3.49 vs. 30.25 ± 4.66	28.6 ± 5.08 vs. 26.4 ± 3.32
Lee et al. (32)	Rotterdam	\	\
Umayal et al. (33)	\	\	\

**Table:** 

**PCOS number**	**Control number**	**Assay for kisspeptin**	**Kisspeptin level in PCOS**	**Kisspeptin level in control**
250	150	Cusabio	1.92 ± 1.29 ng/mL	1.49 ± 1.46 ng/mL
28	30	Phoenix	0.43 ± 0.15 pg/mL	0.39 ± 0.07 pg/mL
19	20	Phoenix	0.24 ± 0.15 pmoL/L^*^	0.19 ± 0.21 pmoL/L
44	44	Phoenix	0.39 ± 0.08 fmoL/mL	0.39 ± 0.07 fmoL/mL
60	57	Mybiosource	5.76 ± 2.11 ng/mL^*^	4.65 ± 2.16 ng/mL
33	36	Phoenix	10.98 ± 6.4 pmoL/L^*^	6.51 ± 3.13 pmoL/L
87	42	Cusabio	0.23 ± 0.20 ng/mL^*^	0.16 ± 0.01 ng/mL
28	13	Phoenix	0.24 ± 0.02 pmoL/L^*^	0.33 ± 0.02 pmoL/L
83	66	Phoenix	2.02 (0.56–4.43) ng/mL${}_{\text{\#}}^{*}$	1.16 (0.45–2.00) ng/mL
29	27	Phoenix	525.49 ± 164.17 pg/mL^*^	354.313 ± 111.38
54	41	/	10.55 ± 6.14 ng/ml^*^	6.98 ± 3.57 ng/mL
55	110	Phoenix	4.873 ± 0.238 nmol/L^*^	4.127 ± 0.132 nmol/L

Values are expressed as the mean ± standard deviation, except for the item marked with #, whose values are expressed as the median (range);

* *indicates a significant difference; PCOS, Polycystic ovary syndrome; BMI, Body mass index; \, Data could not be found in conference articles and unpublished data*.

## Kisspeptin and Metabolic Profiles in Women With PCOS

Kisspeptin was reported to be an effective stimulator of pulsatile GnRH release, which predominantly determines circulating levels of LH. Thus, it is not surprising to find a positive correlation between kisspeptin and LH concentration. However, the majority of studies failed to provide adequate statistical evidence to support this theory. Although Gorkem et al observed a higher concentration of kisspeptin in PCOS women compared with their non-PCOS counterparts (23), kisspeptin and LH levels were not correlated, in line with two other small observational studies (28, 29). In addition, while several investigators noticed a positive correlation between kisspeptin level and LH, they failed to find a significantly higher kisspeptin level in PCOS women compared with control group (25, 26). A recent study may help explain this phenomenon by providing compelling evidence that in patients with PCOS, spontaneous episodic kisspeptin secretion was coupled with LH pulses only in patients without oligomenorrhoea (34). Patients with oligomenorrhea did not demonstrate coupling of kisspeptin pulses with LH pulsatility, suggesting that kisspeptin's regulation of the HPG-axis had been disturbed already. Therefore, due to the heterogeneous constitution of patients with/without oligomenorrhea in different studies, the relationship between kisspeptin and LH secretion is not clear.

Insulin resistance (IR) is found in 50 to 70% of women with PCOS (35), which is widely accepted as a significant risk factor for developing the metabolic syndrome. However, the interaction between kisspeptin and IR in PCOS patients has not been clarified yet. Therefore, in this review, we summarized the existing evidence which is conflicting in terms of the relationship between kisspeptin and IR. Although two cell-based studies found that kisspeptin could inhibit insulin secretion (36, 37), Bowe et al did not demonstrate any change in insulin secretion after kisspeptin was administered (38). *In vitro* studies have demonstrated that kisspeptin facilitated the secretion of glucose-induced insulin in human, porcine, murine islets (39–41). Recently, Izzi-Engbeaya et al demonstrated a beneficial role of kisspeptin in insulin secretion in humans *in vivo* for the first time, which has significant implications for our understanding of the relationship between metabolism and reproduction (42). In women with PCOS, Nyagolova et al. noticed a positive correlation between kisspeptin levels and homeostatic model assessment (HOMA)-IR as well as fasting insulin concentrations in obese participants, which is consistent with a previous Chinese study reporting that two-hour glucose concentrations are positively correlated with kisspeptin levels (22). Nonetheless, another study found that fasting insulin concentration and HOMA-IR were negatively correlated with kisspeptin, meanwhile, glucose/insulin ratio turned out to be positively associated with kisspeptin level (24). Overall, limited and conflicted information is provided in the literature for us to claim an unequivocal conclusion pertaining to the relationship between kisspeptin and IR in general population, let alone in PCOS patients. Due to the heterogeneity of PCOS, future studies should be designed to investigate the dynamic interaction between kisspeptin and glucose tolerance based on different subtypes of PCOS.

Several other metabolic markers, the plasma concentration of testosterone, dehydroepiandrosterone sulfate, free androgen index, leptin, retinol-binding protein, high density lipoprotein and sex hormone binding globulin, have been reported to be positively correlated with kisspeptin at least once and summarized in Table 2. It seems that kisspeptin level is not associated with BMI because only one study found that it is negatively correlated with kisspeptin in logistic regression analysis (24).

**Table 2:** The relationship between kisspeptin and metabolic profiles in PCOS women.

**Reference**	**Sample size**	**Kisspeptin and LH**	**Insulin resistance and kisspeptin**	**BMI and kisspeptin**	**Other metabolic profile**
Emekci Ozay, et al. (26)	250	(+)	HOMA-IR, blood glucose, insulin, (NA)	NA	Leptin(+)
Albalawi et al. (25)	28	(+)	\	NA	\
Chen et al. (22)	19	(+)	2 h glucose(+), HOMA-IR, fasting glucose, fasting insulin, 2 h insulin (–)	NA	T levels(+)
Daghestani (27)	44	NA	Insulin, fasting glucose(–)	NA	HDL(+); TG(–)
Gorkem et al. (23)	60	NA	\	NA	DHEAS, TT(+); FSH(–)
Jeon et al. (28)	33	NA	HOMA-IR, glucose, insulin, glucose/insulin ratio (–)	NA	RBP4(+)
Nyagolova et al. (29)	87	NA	BMI <25: HOMA-IR, insulin(NA); Fasting glucose(–); BMI>25: HOMA-IR, fasting insulin(+);	\	BMI>25: age, T, SHBG, and FAI(+)
Panidis et al. (24)	28	NA	HOMA-IR, fasting insulin(–); Glucose/insulin ratio(+)	(–)	FAI(–); SHBG(+)
Yilmaz et al. (30)	83	(+)	HOMA-IR, fasting glucose, fasting insulin(NA)	NA	DHEA-SO4T, FAI, mFG-score(+); SHBG(–)

*(+), Positive correlation; (–), Negative correlation; NA, No association; \, Not mentioned; HOMA-IR, Homeostasis model assessment – Insulin resistance; T, Testosterone; TT, Total testosterone; HDL, High density lipoprotein; TG, Triglyceride; DHEAS, Dehydroepiandrosterone sulfate; FSH, Follicular stimulating hormone; RBP, Retinol-binding protein; SHBG, Sex hormone binding globulin; FAI, Free androgen index; mFG—score, modified Ferriman–Gallwey score*.

## Kisspeptin Expression in Animal Models of PCOS

Two main kisspeptin neuron populations have been described in rodents, which are located in the hypothalamic ARC and AVPV (43). Kisspeptin neurons in the ARC nucleus are considered a more important kisspeptin generator than those in the AVPV of rodents, which are called KND-y neurons. These neurons autosynaptically regulate pulsatile secretion of kisspeptin through neurokinin B receptors and kappa opioid peptide receptors (44). Although some evidence suggested that kesspeptin matters in the physiological regulation of HPG axis (45, 46), the function of KISS1 system in the pathogenesis of PCOS is still unclear. Recently, several studies measured the expression of the KISS1 gene and kisspeptin immunoreactivity in PCOS rat model (shown in Table 3), suggesting that the KISS1 system acts differently in various PCOS models. Brown et al. found that hypothalamic KISS1 mRNA and kisspeptin immunoreactivity is reduced in DHT(dihydrotestosterone)-induced PCOS rat models, which was the first attempt to note an androgenic effect on hypothalamic KISS1 in female rats (48). Subsequently, down-regulated expression of the KISS1 gene was also detected in two rat models induced by testosterone and estradiol respectively. The KISS1 receptor gene, however, was only upregulated in the estradiol-induced model (50). In contrast, a recent study failed to find a difference in kisspeptin-immunoreactivity in postnatal DHT-induced rats compared with controls as observed in the study conducted by Brown et al. However, they observed increased KISS1 expression in the prenatal DHT-induced PCOS model (52). Two letrozole-induced PCOS rat models, featuring irregular cycles, elevated serum LH and testosterone levels presented new findings. The authors noticed more kisspeptin-positive cells in the ARC nucleus but less in the AVPV nucleus (47). The other study also found kisspeptin mRNA expression increased in the ARC instead of the AVPV, which supports a theory that the ARC serves as a more important regulator of KISS1 system in the PCOS animal model (51). In addition, increased hypothalamic kisspeptin-positive cells were also reported in a RU486 (mifepristone)-induced rat (49). Therefore, animal models of PCOS with distinct metabolic phenotypes may result in varying kisspeptin expression in different parts of the hypothalamus. In this review, an interesting conclusion is that the KISS1 system is over-activated only in the animal models with elevated LH levels, in line with the presumed function of kisspeptin in the regulation of the HPG-axis. Besides, kisspeptin immunoreactivity is decreased in the PCOS rats with higher weight, which suggested that body weight is negatively correlated with the activity of KISS1 system. In conclusion, findings from animal models suggested that the KISS1 system only upregulated in the PCOS phenotypes with higher LH levels and normal body weight.

**Table 3:** Kisspeptin expression in PCOS animal models.

**Reference**	**PCOS model**	**Model characteristics**	**Main conclusion**
Aliabadi et al. (47)	Letrozole-induced	Irregular diestrus; Persistent anovulation; Polycystic ovary; Testosterone(↑) and LH(↑); EG(↓)	In ARC nucleus, kisspeptin - positive cells (↑); In AVPV nucleus, kisspeptin-positive cells(↓)
Brown et al. (48)	DHT-induced	Body weight(↑); Insulin resistance	Hypothalamic KISS1 mRNA and kisspeptin immunoreactivity (↓)
Kondo et al. (49)	RU486 exposure	Irregular cycles; Testosterone(↑) and LH(↑)	Hypothalamus kisspeptin-positive cells (↑)
Marcondes et al. (50)	Neonatal exposure of EG/TG	EG: anovulation and Polycystic ovaries; LH(–), Testosterone(–); TG: LH (↑) and Testosterone (↑); Anovulation; Polycystic ovaries	EG: KISS1(↓), KISS1r (↑) TG: KISS1(↓)
Matsuzaki et al. (51)	Letrozole-induced	Irregular diestrus; Persistent anovulation; Polycystic ovary; Testosterone(↑) and LH(↑); EG(↓)	In ARC: Kisspeptin mRNA expression(↑)
Kondo et al. (49)	1.Postnatal DHT exposure 2.Prenatal DHT exposure	Body weight (↑); Persistent diestrus; LH(–); Irregular diestrus; LH(↑) Body weight(–)	1. In ARC, kisspeptin immunoreactivity (–) 2. In ARC, kisspeptin/NKB immunoreactivity (↑)

*(↑), The index is elevated compared with control group; (↓), The index is decreased compared with control group; (–), The index is comparable with control group; PCOS, Polycystic ovary syndrome; DHT, Dihydrotestosterone; AVPV, Anteroventral periventricular nucleus; ARC, Arcuate nucleus; RU486, mifepristone, 11β-(4-dimethylamino)ohenyl-17β-hydroxy-17-(1-propynyl)estra-4,9-dien-3-one; EG, Estradiol group; TG, Testosterone group; qRT—PCR, Quantitative real-time polymerase chain reaction; NKB, neurokinin B*.

## Conclusion

Overall, kisspeptin levels are higher in the PCOS population, which supports the hypothesis that an over-active KISS1 system causes HPG-axis overactivity, leading to irregular menstrual cycles and excessive androgen release. However, findings from animal studies suggest that kisspeptin levels are not increased in all subtypes of PCOS. In this review, although we conclude that kisspeptin concentration is elevated in the subtypes of PCOS featured with higher LH levels and normal body weight, some of the changes in animal models reflect more about the way the “PCOS model” is generated rather than unveiling too much pathophysiologic change about PCOS in humans. Future studies investigating the relationships between kisspeptin and other metabolic factors such as LH and IR, which are still unclear, would be warranted.

## Author Contributions

RT and XD were engaged in analysis of data, prepared, and drafted manuscript. JZ contributed to conception, study design, and article revision.

### Conflict of Interest Statement

The authors declare that the research was conducted in the absence of any commercial or financial relationships that could be construed as a potential conflict of interest.
